# Incidence of surgically treated post-traumatic hydrocephalus 6 months following head injury in patients undergoing acute head computed tomography

**DOI:** 10.1007/s00701-022-05299-3

**Published:** 2022-07-07

**Authors:** Aaro Heinonen, Minna Rauhala, Harri Isokuortti, Anneli Kataja, Milaja Nikula, Juha Öhman, Grant L. Iverson, Teemu Luoto

**Affiliations:** 1 The Faculty of Medicine and Health Technology, Tampere University, Arvo Ylpön katu 34, 33520 Tampere, Finland; 2 Department of Neurosurgery, Tampere University Hospital, Tampere, Finland; 3 Department of Neurosurgery, Helsinki University Hospital and University of Helsinki, Helsinki, Finland; 4 Department of Radiology, Medical Imaging Centre, Tampere University Hospital, Tampere, Finland; 5 Department of Physical Medicine and Rehabilitation, Harvard Medical School, Boston, MA USA; 6 Department of Physical Medicine and Rehabilitation, Spaulding Rehabilitation Hospital and Spaulding Research Institute, Charlestown, MA USA

**Keywords:** Post-traumatic hydrocephalus, Traumatic brain injury, Ventriculoperitoneal shunt, Neurosurgery

## Abstract

**Background:**

Post-traumatic hydrocephalus (PTH) is a well-known complication of head injury. The percentage of patients experiencing PTH in trauma cohorts (0.7–51.4%) varies greatly in the prior literature depending on the study population and applied diagnostic criteria. The objective was to determine the incidence of surgically treated PTH in a consecutive series of patients undergoing acute head computed tomography (CT) following injury.

**Methods:**

All patients (*N* = 2908) with head injuries who underwent head CT and were treated at the Tampere University Hospital’s Emergency Department (August 2010–July 2012) were retrospectively evaluated from patient medical records. This study focused on adults (18 years or older) who were residents of the Pirkanmaa region at the time of injury and were clinically evaluated and scanned with head CT at the Tampere University Hospital’s emergency department within 48 h after injury (*n* = 1941). A thorough review of records for neurological signs and symptoms of hydrocephalus was conducted for all patients having a radiological suspicion of hydrocephalus. The diagnosis of PTH was based on clinical and radiological signs of the condition within 6 months following injury. The main outcome was surgical treatment for PTH. Clinical evidence of shunt responsiveness was required to confirm the diagnosis of PTH.

**Results:**

The incidence of surgically treated PTH was 0.15% (*n* = 3). Incidence was 0.08% among patients with mild traumatic brain injury (TBI) and 1.1% among those with moderate to severe TBI. All the patients who developed PTH underwent neurosurgery during the initial hospitalization due to the head injury. The incidence of PTH among patients who underwent neurosurgery for acute traumatic intracranial lesions was 2.7%.

**Conclusion:**

The overall incidence of surgically treated PTH was extremely low (0.15%) in our cohort. Analyses of risk factors and the evaluation of temporal profiles could not be undertaken due to the extremely small number of cases.

## Introduction

Traumatic brain injury (TBI) is a significant cause of both mortality and morbidity [8]. Post-traumatic hydrocephalus (PTH) is a well-known complication of TBI [31]. PTH signs and symptoms (e.g., headache, nausea, cognitive dysfunction, ataxia, obtundation, a tetrad of psychomotor retardation, memory loss, gait trouble, and urinary incontinence [1, 33]) overlap with the signs and symptoms of the primary injury, which makes the condition difficult to diagnose in some cases [11]. A classic clinical feature to suspect PTH after TBI is poor improvement or stagnation of recovery [11, 32]. Early recognition of PTH is essential in the follow-up of patients with TBIs, as cerebrospinal fluid diversion (ventricular shunting) has been shown to improve outcome during rehabilitation [27, 41].

PTH diagnosis is generally based on a combination of clinical signs, symptoms, and radiological findings [11, 13, 18]. Various criteria for PTH diagnosis have been suggested, but no universal criteria have been established [25]. The percentage of patients experiencing PTH in trauma cohorts varies broadly from 0.7 to 51.4% [2, 4, 11, 31, 42]. The broad range is largely explained by the differences in the study population, applied diagnostic criteria, and the individual study designs [4]. A recent Taiwanese study with a large retrospective cohort (*n* = 23,775) reported a very low incidence of PTH, only 0.48 to 1.98% [11]. In that study, PTH incidence peaked within three months after injury. Those patients with subarachnoid hemorrhage (SAH) had a threefold risk of developing PTH compared to patients with no SAH during the 2-year follow-up period. Earlier studies with lower numbers of cases (*n* = 139–444) are inconsistent regarding SAH as a risk factor for PTH [24, 25, 38]. Also, decompressive craniectomy [13, 17] and the presence of subdural hygroma after craniectomy [29] seem to increase the risk for PTH. In craniectomy, the proximity of the craniotomy to the skull midline [4, 43] might increase the risk as well. Other possible risk factors for PTH increased age, cerebrospinal fluid infection, and intraventricular hemorrhage [4, 13]. Inflammation-mediated adhesions related to intracranial hemorrhages can explain the predisposition to PTH [36]. In children, PTH incidence also varies greatly depending on study population and applied diagnostic criteria. However, pediatric PTH incidence is suspected to be lower compared to adults [16, 35, 40]. Similar to adults, severe TBI, decompressive craniectomy, and SAH have been reported to increase the risk for PTH in children [9, 40]. Additionally, unique pediatric PTH risk factors include young age, electrolyte disorder, and weight loss [7, 9, 35].

As a relatively rare condition with signs and symptoms that are sometimes elusive or difficult to differentiate from the primary injury, PTH remains a diagnostic challenge. The literature regarding PTH is limited. It has been shown that the treatment of PTH is beneficial [41]. The objective of this study was to determine the incidence of surgically treated PTH within 6 months from head injury in a series of consecutive adult patients undergoing acute head CT following injury. A specific interest was to document the time interval between the injury and the manifestation of PTH. We also aimed to determine if there are any identifiable pre- or peri-injury risk factors for surgically treated PTH.

## Methods and materials

### Material and ethics

This study is a part of the Tampere Traumatic Head and Brain Injury Study. All consecutive patients with head injuries who underwent acute CT, treated at the Tampere University Hospital’s Emergency Department between August 2010 and July 2012, were retrospectively evaluated from hospital’s patient records. There was a total of 3023 head injuries in 2908 patients during this 2-year period. Data collection included a 6-month follow-up period for PTH. The length of the follow-up period was based on the most likely occurrence time of PTH reported in prior studies [11, 19, 25, 31, 42].

**Fig. 1:**
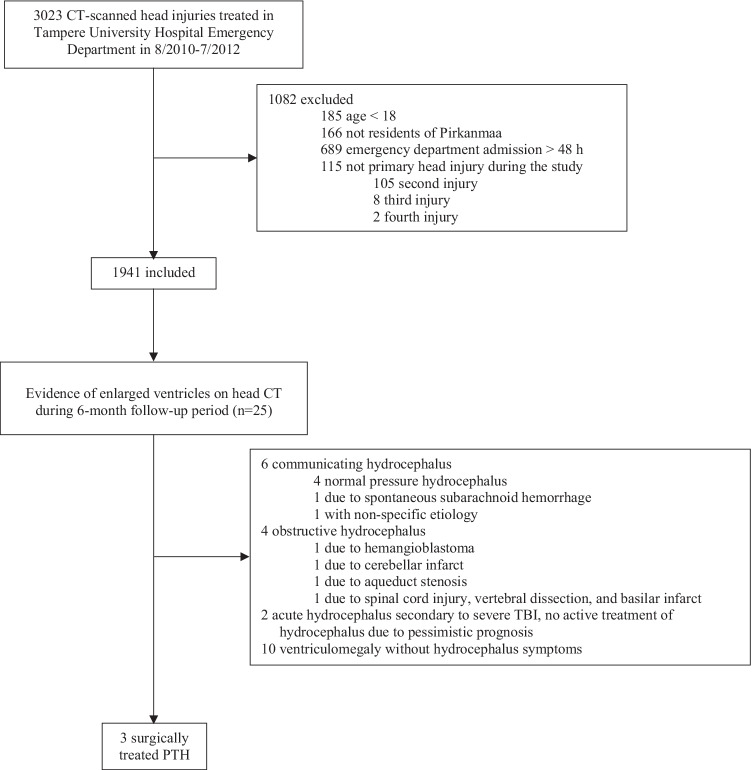
Study profile. *CT* computed tomography, *TBI* traumatic brain injury, *PTH* post-traumatic hydrocephalus

This study focused on adult (18 years or older) patients who were residents of the Pirkanmaa region at the time of injury and were clinically evaluated and scanned with head CT at the Tampere University Hospital’s emergency department within 48 h (≤ 48 h) after head injury. Patients who had suffered more than one head injury during the study period were included once in the study sample with the initial head injury as the index injury. A total of 1941 adult patients undergoing acute head CT following injury were identified. A flowchart of the study sample is provided in Fig. 1.

The Pirkanmaa region is a geographically well-defined area with both rural and urban areas that holds one of Finland’s five university hospitals with a neurosurgical service (Tampere University Hospital, Tampere, Finland). During the study period, Pirkanmaa had 490,000 residents, which comprised 9% of the total population of Finland (5.4 million) at the time. In addition to Tampere University Hospital, there is one local hospital with a CT scanner used for patients with head injuries in the Pirkanmaa region. However, most of the head trauma patients, and all the patients requiring neurosurgical care, are evaluated at the Tampere University Hospital.

The study was approved by the Ethics Committee of the Pirkanmaa Hospital District, Tampere, Finland (ethical code: R10027). All data was collected retrospectively without contacting the patients; therefore, no written informed consent was obtained or required.

### Data collection

A detailed and structured data collection was performed from the medical records. Two neuroradiologists examined all the head CT scans. Study data were collected before the National Institution of Neurological Disorders and Stroke Common Data Elements (CDEs) [15] for TBI imaging were established. However, all CDEs possible with noncontrast structural CT were included [23]. Variables collected included demographics, antithrombotic medication (including anticoagulants and antiplatelets), injury-related information, clinical TBI indices, emergency head CT findings (acute traumatic lesions), acute neurosurgery due to TBI, and follow-up findings in relation to PTH. Minimal criteria for TBI were based on the World Health Organization’s (WHO) definition [10]. Cases with Glasgow Coma Scale (GCS) score < 13 after 30-min postinjury, post-traumatic amnesia more than 24 h, and/or loss of consciousness more than 30 min were coded as moderate to severe TBI. There was a considerable number of patients with missing GCS scores. Based on other clinical findings and examinations reported in the patient medical records, and the lack of reported low GCS or other sign of more severe TBI, these patients were coded as having a mild TBI or no TBI.

A thorough review of records for neurological signs and symptoms of hydrocephalus was conducted for all patients having a radiological suspicion of hydrocephalus/ventriculomegaly. The diagnosis of PTH was based on clinical and radiological signs of the condition. The main outcome variable was specified as *surgically treated post-traumatic hydrocephalus* to rule out conditions not requiring treatment, such as post-traumatic ventriculomegaly resulting from secondary atrophy. Additionally, clinical evidence of shunt responsiveness (i.e., improvement in preoperative PTH signs and symptoms) was required. Patients with other apparent causes for hydrocephalus (e.g., normal pressure hydrocephalus diagnosis prior to injury and obstructive hydrocephalus due to posterior fossa lesions) were considered to have non-traumatic hydrocephalus.

### Statistical analysis

IBM SPSS Statistics for Windows (version 27, IBM Corp.) was used for data analyses. We used Kolmogorov–Smirnov test to examine variable distribution. Descriptive statistics (frequency, percentage, median, interquartile range (IQR)) were used to describe variable characteristics.

### Data availability statement

The data that support the findings of this study are available from the corresponding author upon reasonable request.

## Results

### Characteristics of the cohort

The cohort included 1941 patients with the median age of 59 years. Of the 1941 patients, 1122 (58%) were men. The most common mechanism of injury was a ground-level fall (GLF) in 1016 (52%) patients. Alcohol intoxication was reported for 567 (29%) patients and 494 (26%) patients were on antithrombotic medication at the time of injury. Loss of consciousness was documented in 417 (22%) patients and amnesia in 510 (26%) patients. GCS scores were not reported for a third of the patients (*n* = 663, 34%). Among patients with reported GCS scores, the scores were distributed as follows: 57% (*n* = 1100) arrived at the emergency department with a GCS score of 13–15, 5.0% (*n* = 97) of patients with a GCS score of 9–12, and 4.2% (*n* = 81) of patients with a GCS score of 3–8. The majority of the patients, 1269 (65%), had a mild TBI. Moderate to severe TBI was reported for 187 (9.6%) patients, and 485 (25%) patients did not have evidence of TBI in their records. The most frequent acute traumatic lesions on admission head CT were a subdural hematoma (acute and/or chronic) in 254 (13%) patients and subarachnoid hemorrhage in 216 (11%). Emergency trauma neurosurgery was performed on 111 (5.7%) patients. Characteristics of the study cohort are summarized in Table 1.

**Table 1:** Characteristics of the study cohort (*n* = 1941)

Variable		
Age median, years (IQR)	58.7 (39.1–58.7)	
	***n***	**%**
Men	1122	57.8
Cause of injury		
Ground-level fall	1016	52.3
Motor vehicle accident	261	13.4
Fall from a height	215	11.1
Traffic accident as pedestrian or bicyclist	116	6.0
Other	333	17.2
Alcohol intoxication	567	29.2
Antithrombotic medication	494	25.5
Loss of consciousness	417	21.5
Amnesia	510	26.3
Glasgow Coma Scale		
13–15 points	1100	56.7
9–12 points	97	5.0
3–8 point	81	4.2
Unknown	663	34.2
Traumatic brain injury severity		
No traumatic brain injury	485	25.0
Mild	1269	65.4
Moderate to severe	187	9.6
Acute traumatic lesion on head CT-scan		
Subdural hematoma (acute and/or chronic)	254	13.1
Subarachnoid hemorrhage	216	11.1
Contusion	152	7.8
Intraventricular hemorrhage	54	2.8
Epidural hematoma	14	0.7
Diffuse axonal injury	7	0.4
Emergency neurosurgery due to acute traumatic brain injury	111	5.7
Decompressive craniectomy	14	0.7
Shunted post-traumatic hydrocephalus within 6-month post-injury	3	0.15

*IQR*, interquartile range; *CT*, computed tomography

### Post-traumatic hydrocephalus

Only three patients were diagnosed and surgically treated for clinically and radiologically diagnosed PTH during the 6-month follow-up period. A ventriculoperitoneal shunt was placed in all of these patients. The three patients were reported to benefit from the shunt, but there was no systematic follow-up after the PTH diagnosis and surgical treatment. A positive response to shunt surgery was based on the information reviewed from the patient records. If there was consistent clinical improvement after the shunt operation and no other distinct cause for it, the patient was considered to benefit from the shunt. As a result, the incidence of surgically treated PTH in our cohort was 0.15% (Table 1). One of the surgically treated PTH patients had a mild TBI, and thus, the incidence of PTH among mild TBI patients was 0.08% (1 per 1269). Two of the three patients had a moderate TBI, and the incidence of PTH among moderate to severe TBI patients was 1.1% (2 per 187). All the surgically treated PTH cases underwent acute neurosurgery due to the index head trauma. The incidence of surgically treated PTH among patients undergoing acute neurosurgery was 2.7% (3 per 111).

Of the three patients with PTH, two were men aged 56 and 87 years, and one was a 62-year-old woman. Both men sustained a moderate TBI and the woman sustained a mild TBI. GLF was the injury mechanism for all three patients. The 87-year-old man was on antithrombotic medication (warfarin) at the time of trauma. Alcohol intoxication was reported for the 56-year-old man and for the 62-year-old woman. Acute traumatic lesions were seen in all three patients: contusions and acute subdural hematoma (SDH) in all patients and SAH in two patients (both of the men). All three patients underwent acute neurosurgery during the initial admission. The 56-year-old man underwent evacuation of intracranial contusion hemorrhage and SDH through craniotomy. An acute SDH craniotomy was performed on the 87-year-old man. An emergency trepanation and evacuation of a subdural hygroma were performed on the woman. The surgically treated PTH patients had 2, 5, and 5 months of delay between the index injury and the shunt surgery. A detailed description of all patients with surgically treated PTH within 6 months after injury is presented in Table 2. Due to the small number of cases (*n* = 3), group analyses on possible PTH risk factors were not performed.

**Table 2:** Detailed description of patients with post-traumatic hydrocephalus within six months after injury (*n* = 3)

Patient	Injury mechanism	TBI severity	CT findings	Acute neurosurgery	Hydrocephalus symptoms	Shunt surgery; delay since injury	Hydrocephalus etiology	Clinical improvement after shunt placement
Woman, age 62	Fall	Mild	Contusion, acute SDH, subdural hygroma	TRP, evacuation of subdural hygroma	Headache, nausea, vertigo	VP, 2 months	PTH	Yes
Man, age 56	Fall	Moderate	Skull fracture, SAH, contusion, acute SDH	CRT, evacuation of ICH and SDH	Disorientation, slowed down	VP, 5 months	PTH	Yes
Man, age 87	Fall	Moderate	SAH, contusion, acute SDH	CRT, evacuation of acute SDH	General malaise	VP, 5 months	PTH	Yes

All mechanisms of injury were ground-level falls. *TBI*, traumatic brain injury; *CT*, computed tomography; *SAH*, subarachnoid hemorrhage; *SDH*, subdural hemorrhage; *CRT*, craniotomy; *ICH*, intracerebral hemorrhage; *PTH*, post-traumatic hydrocephalus; *TRP*, burr hole trepanation; *VP*, ventriculo-peritoneal

### Other hydrocephalus after head trauma

Two patients among our cohort of 1941 patients experienced acute hydrocephalus secondary to severe TBI, and their initial CT scan revealed enlarged ventricles. These two patients underwent acute neurosurgery because of their severe TBI, and they initially had a very poor prognosis. During the initial hospital admission, both of these patients died due to complications related to the severe TBI. The active treatment of hydrocephalus in these moribund cases was considered as disadvantageous and no surgical interventions for hydrocephalus were performed.

In addition, 10 cases of non-traumatic hydrocephalus who underwent neurosurgery were recognized either on the first head CT or during the follow-up period. These hydrocephalus cases were categorized as obstructive hydrocephalus (*n* = 4) and communicating hydrocephalus (*n* = 6). The etiologies for obstructive hydrocephalus cases were hemangioblastoma, cerebellar infarct, aqueduct stenosis, and cervical spinal cord injury with vertebral dissection and basilar infarct. The communicating hydrocephalus cases were due to normal pressure hydrocephalus (*n* = 4), spontaneous SAH, and one with a non-specific etiology. The six patients with communicating hydrocephalus had pre-existing symptoms and enlarged ventricles before or at the time of head trauma.

## Discussion

### Summary of the key findings

The incidence of surgically treated PTH among our cohort of 1941 patients undergoing acute head CT was 0.15%. The incidence was 0.08% among patients with mild TBI and 1.1% among patients with moderate to severe TBI. All three patients who developed PTH underwent acute neurosurgery for intracranial bleeding due to the index injury. The incidence of surgically treated PTH among patients undergoing acute neurosurgery was 2.7%. No patients who did not undergo acute neurosurgery due to head trauma developed surgically treated PTH. Our three PTH patients underwent ventriculoperitoneal shunt surgery within 2 to 5 months following injury.

### Comparison of the current findings to prior literature

The literature on PTH is limited. In our study, PTH incidence was lower than previously reported. Earlier studies show percentages of patients experiencing PTH ranging from 0.7 to 51.4% [2, 4, 11, 31, 42]. A recent Taiwanese study with a large retrospective cohort (*n* = 23,775) reported PTH incidence of 0.48% for patients without traumatic SAH and 1.98% for patients with traumatic SAH [11]. In that study, patients with all severities of TBI were included. PTH occurrence was the highest during the first 3 months after head trauma, but the exact time from injury to PTH occurrence was not reported. Wettervik et al. [42] reported that 3.5% experienced PTH among patients treated in neurointensive care unit (*n* = 836).

We found three studies that described the temporal profile of PTH. Mazzini et al. [31] studied 140 patients with severe TBIs, with the mean time from injury to shunt surgery being 55 days (45% experienced PTH). Kammersgaard et al. [25] had a similar result. Among 444 patients with severe TBIs, more than 75% of cases that developed PTH, PTH occurred within 8 weeks (56 days) from the index injury (14% had PTH in that study). Wetterwik et al. [42] reported a median time of 5 months from injury to shunt surgery in a cohort of 836 patients treated in neurointensive care (3.5% experienced PTH).

The percentages of people with PTH, across studies, varies widely for several reasons, such as differences in sample sizes and in inclusion and exclusion criteria. Selection bias can occur when subgroups of patients with varying prevalence of factors predisposing to PTH are studied. There are several guidelines on the use of head CT for patients with acute head injury [20, 37, 39]. In clinical practice, patients are scanned if certain risk factors for traumatic intracranial lesions are evident. Of course, not all patients with a minor head injury are imaged. We excluded patients with head trauma who were not scanned during the first 48 h after injury. Consequently, PTH incidence among all patients presenting to the emergency department following head injury is probably even lower than in our study.

It is important to note that the applied diagnostic criteria for PTH differs between studies [4]. PTH diagnosis is generally based on clinical presentation and radiological findings [11, 13]. A more reliable diagnosis is established with inclusion of shunt responsiveness into the diagnostic criteria [32]. Additionally, register studies may include other types of treated hydrocephalus after head trauma separate from PTH. Ventriculomegaly after severe TBI is a relatively common finding [31]. Difficulty distinguishing PTH from atrophy, especially in patients with ambiguous clinical presentation, has led to a search for new diagnostic methods [28, 30, 34, 44]. To date, no universal criteria for PTH have been established [25]. Our study was based on patients with PTH who benefited from shunt surgery and non-traumatic hydrocephalus cases were excluded by careful evaluation of medical records and head CT scans. Another factor that complicated comparing our results to previous studies was the use of term “incidence” in the prior literature. Not all studies we examined reported clearly whether they reported true incidence or only the percentage or number of patients experiencing PTH in a trauma cohort.

We were not able to conduct statistical analyses to examine pre-operative and perioperative risk factors for surgically treated PTH given there were only three cases. Interestingly, however, all our head trauma patients, who developed surgically treated PTH, underwent acute neurosurgery for intracranial bleeding. In the previous literature, increased age, intraventricular hemorrhage, subarachnoid hemorrhage, cerebrospinal fluid infection, decompressive craniectomy, and the presence of subdural hygroma have been reported to predispose to PTH [4, 13, 24, 29, 38]. All of our three patients who developed surgically treated PTH had at least one of these aforementioned risk factors: SAH, subdural hygroma, or increased age. None of the patients in our cohort who underwent decompressive craniectomy (*n* = 14) developed PTH in the 6-month follow-up period, although this surgery is considered an independent risk factor for PTH [4, 12, 22, 24]. The percentages of patients experiencing PTH after decompressive craniectomy due to a head injury are 26–30% in previous studies [5, 21, 26].

### Strengths and limitations

Our study represents an extensive retrospective series of consecutive patients from one geographically well-defined area. All the patients were evaluated and scanned in the emergency department of one university hospital. Although our study was not population-based, it reflects the incidence of surgically treated PTH in head trauma patients in Pirkanmaa, Finland. The results are generalizable for populations similar to Finland, with an aging population and GLF as the main cause of TBI [8].

Our study has several limitations. Due to the retrospective design, not all desired data were available. The lack of a systematic long-term follow-up protocol for all of our TBI patients means that some cases of PTH could have been missed. The patients were followed up at different time points postinjury on a purely clinical basis. The majority of patients were evaluated by a neurosurgeon only if neurosurgically treatable problems were suspected. Therefore, this follow-up practice might have increased the probability of missing PTH cases. However, we estimate the rate of missed cases to be low because TBI patients, family members, and healthcare professional are informed to contact the neurosurgical department if new worrisome signs or symptoms emerge after TBI. In addition, no routine outpatient clinic follow-up was performed with the PTH patients. Another limitation of our study is that the number of cases was too small for PTH risk factor analyses or meaningful measurements of the temporal profile of PTH development, and thus, we could not address all our a priori research questions.

### Directions for future research

New examination methods might help improve the diagnosis of PTH, when the diagnosis is unclear based on clinical assessment and routine radiological findings. Future applications of diffusion tensor imaging might be useful in distinguishing true hydrocephalus from ventriculomegaly [34]. Additionally, studies on cerebrospinal fluid (CSF) dynamics [3, 28, 30] have shown that parameters such as resistance to CSF outflow and pulse amplitude of intracranial pressure might be useful in selecting patients with PTH who may benefit from ventriculoperitoneal shunting [34]. Blood-based biomarkers (e.g., S100B) [44] also show promise for predicting the development of PTH. Chen et al. [12] introduced a risk scoring system based on clinical characteristics to predict PTH after TBI with promising results. Endoscopic third ventriculostomy might be a useful treatment option for PTH, even though this intervention has been considered contraindicated for PTH in the past [6, 14].

Literature about PTH is relatively scarce. Uniform diagnostic criteria for PTH in future studies would facilitate better comparison of scientific findings. More studies with larger patient cohorts are needed to have sufficient number of cases with the primary outcome (PTH) to examine risk factors, temporal development, and optimal treatment modalities. Studies on CSF pressure dynamics and newer imaging modalities might lead to improved diagnostic accuracy in unclear cases.

## Conclusions

The incidence of surgically treated PTH in our cohort was extremely low (overall 0.15%, mild TBI 0.08%, moderate-severe TBI 1.1%). Analyses of risk factors and the evaluation of temporal profiles could not be undertaken due to the extremely small number of cases.

## Data Availability

The data that support the findings of this study are available from the corresponding author upon reasonable request.

## Code availability

The code and analyses used are available from the corresponding author upon reasonable request.
